# Retrosternal hematoma causing torsade de pointes after coronary artery bypass graft surgery; a case report

**DOI:** 10.3389/fcvm.2024.1331873

**Published:** 2024-05-20

**Authors:** Mohammadbagher Sharifkazemi, Mohammad Ghazinour, Mehrzad Lotfi, Soorena Khorshidi, Tahereh Davarpasand

**Affiliations:** ^1^ Department of Cardiology, Nemazee Hospital, Shiraz University of Medical Sciences, Shiraz, Iran; ^2^ Department of Surgery, Shahid Faghihi Hospital, Shiraz University of Medical Sciences, Shiraz, Iran; ^3^ Department of Radiology, Shahid Faghihi Hospital, Shiraz University of Medical Sciences, Shiraz, Iran; ^4^ Department of Cardiology, Tehran Heart Center, Tehran University of Medical Sciences, Tehran, Iran

**Keywords:** myocardial infarction, coronary artery bypass, torsades de pointes, cardiac tamponade, case report

## Abstract

Myocardial infarction is among the top causes of mortality worldwide. Survivors may also experience several complications. Infarct-related torsade de pointes (TdP) is an uncommon complication. In the context of myocardial infarction, coronary artery bypass graft (CABG) surgery is the prevalent therapeutic modality associated with several early and late complications. Ventricular tachyarrhythmias, including TdP, because of electrical inhomogeneity, would potentially be a lethal complication of CABG. Here, we report the occurrence of medically intractable TdP in the presence of an uncommon case of a post-CABG retrosternal hematoma. Arrhythmia was properly resolved after hematoma removal surgically. It showed the possibility of a “cause and effect” relationship between these two complications. This unique case emphasizes the post-CABG medically-resistant TdP, considering the mechanical pressure effect of retrosternal hematoma that stimulates this potentially malignant arrhythmia, especially in the absence of electrolyte disturbances and evident symptoms of ongoing ischemia.

## Introduction

Myocardial infarction (MI), especially ST-elevation MI (STEMI), is among the top causes of mortality worldwide, responsible for at least 15% of mortality each year (1). Although emergency management, especially coronary artery bypass grafting (CABG) and percutaneous intervention, reduced its mortality rate (2), several complications are observed in the survivors, such as post-MI mechanical complications and electrical disturbances (3). Complications are sorted into early and late events, most requiring emergency interventions (4).

Arrhythmias and conduction disturbances, such as ventricular fibrillation (VF), ventricular tachycardia (VT), and many other arrhythmias, are not uncommon complications, both in early and late stages even after CABG that needs medical and sometimes surgical intervention (5). Polymorphic VT in the setting of acute MI is commonly observed; however, acquired long QT syndrome infarct-related torsade de pointes (TdP) is uncommon (6). Because this complication is potentially lethal, paying greater attention to its manifestations and associated clinical signs and symptoms is important for accurate and on-time diagnosis and treatment. Considering the few cases reported in the literature, reporting characteristics and outcomes of new cases can help increase the physician's knowledge about this lethal complication in the post-CABG status. Accordingly, in the present study, we report a drug-resistant TdP in the presence of retrosternal hematoma as an early complication after CABG, which frequently stimulated arrhythmias, and finally stopped after hematoma removal surgically.

## Case presentation

**Figure 1:**
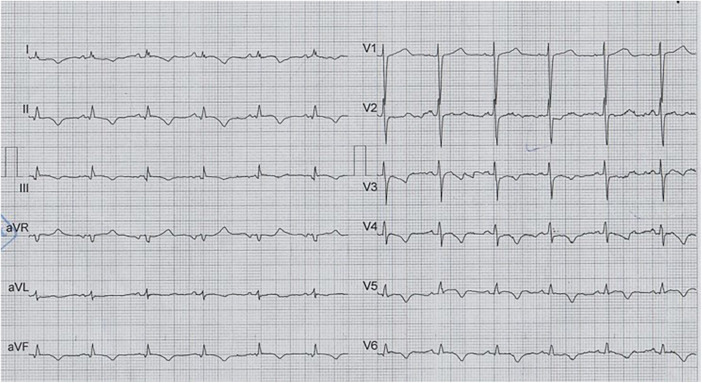
The first ECG on arrival and before CABG, which illustrates normal sinus rhythm with low voltage QRS and prolonged qTcB (505 ms), in addition to findings compatible with inferolateral STEMI.

The patient was a 69-year-old man with a positive history of diabetes mellitus, admitted to the emergency department with the chief complaint of retrosternal pain, accompanied by nausea and dyspnea, for several hours before admission. He was diagnosed with an inferolateral ST-elevation MI, accompanied by a prolonged QTc interval on the first electrocardiogram (ECG) (Figure 1) and transferred to the catheterization laboratory for emergency coronary angiography, which revealed significant stenosis in the terminal end of the left main artery, in addition to advanced three-vessel disease. Accordingly, CABG was recommended.

He was admitted to the cardiac surgery ward for about 10 days to control his blood sugar and become prepared for the surgery. During this period, no ventricular arrhythmia was recorded. The serum levels of potassium, magnesium, and calcium were evaluated, all of which were in the normal range. The follow-up echocardiography showed dilated left ventricle (LV) with global hypokinesia and severe LV dysfunction, LV ejection fraction (EF) of 25%, a huge apical aneurysm, a large semi-mobile apical clot (its area was equal to 2.4 cm^2^), and grade II diastolic dysfunction.

Accordingly, CABG (one arterial and two vein graft: LIMA-LAD, SVG-OM, and SVG-PDA) was done with LV aneurysmectomy, LV clot removal, and intra-aortic balloon pump (IABP) insertion. Aortic clamp time was 50 min, cardiopulmonary bypass (CPB) time was 90 min, and total operation duration was 200 min. Two chest tubes were inserted for the patient in the substernal and left pleural spaces. After the surgery, he was transferred to the cardiac surgery intensive care unit (ICU). The post-CABG ECG of the patient (in ICU) illustrated normal sinus rhythm with low voltage QRS, and bigeminal ventricular extrasystole (PVCs) with short coupling interval (R on T wave; Supplementary Figure S1).

**Figure 2:**
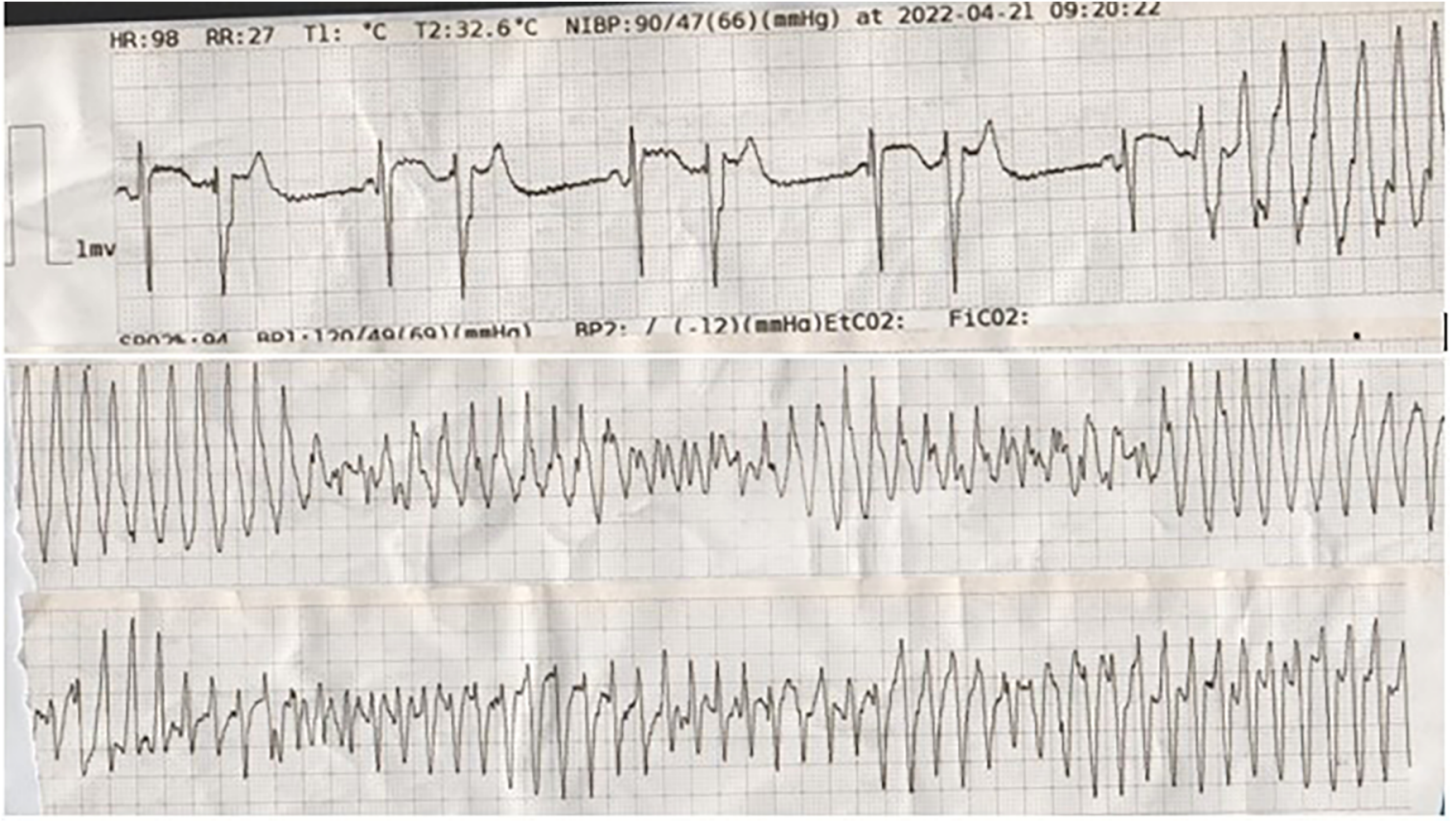
The tracing shows bigeminal PVBs with short coupling intervals (R on T), which degenerated into torsade de pointes.

The day after the surgery, the serum level of hemoglobin was 9 mg/dl, and he was transfused with one unit (400 ml) packed cell. On the same day, anti-platelet therapy started, and IABP was removed (24 h after the surgery). On the first day 600 ml was darianed, and on next day (48 h after the surgery), 100 ml; the chest tube was removed after 48 h. On the same day (2 days after the surgery), he developed recurrent episodes of TdP (Figure 2), one of them degenerated into VF, causing cardiovascular collapse. Hence, he was re-intubated and received 200 J DC shock, in addition to 2 g IV lidocaine plus 24 g IV magnesium sulfate in a 24-h infusion. Moreover, because of persistent low systolic blood pressure, IABP was re-inserted. The serum levels of cardiac enzymes (such as high sensitivity troponin I) and the electrolytes were normal. The patient's LVEF was 25%–30% on the third postoperative day.

**Figure 3:**
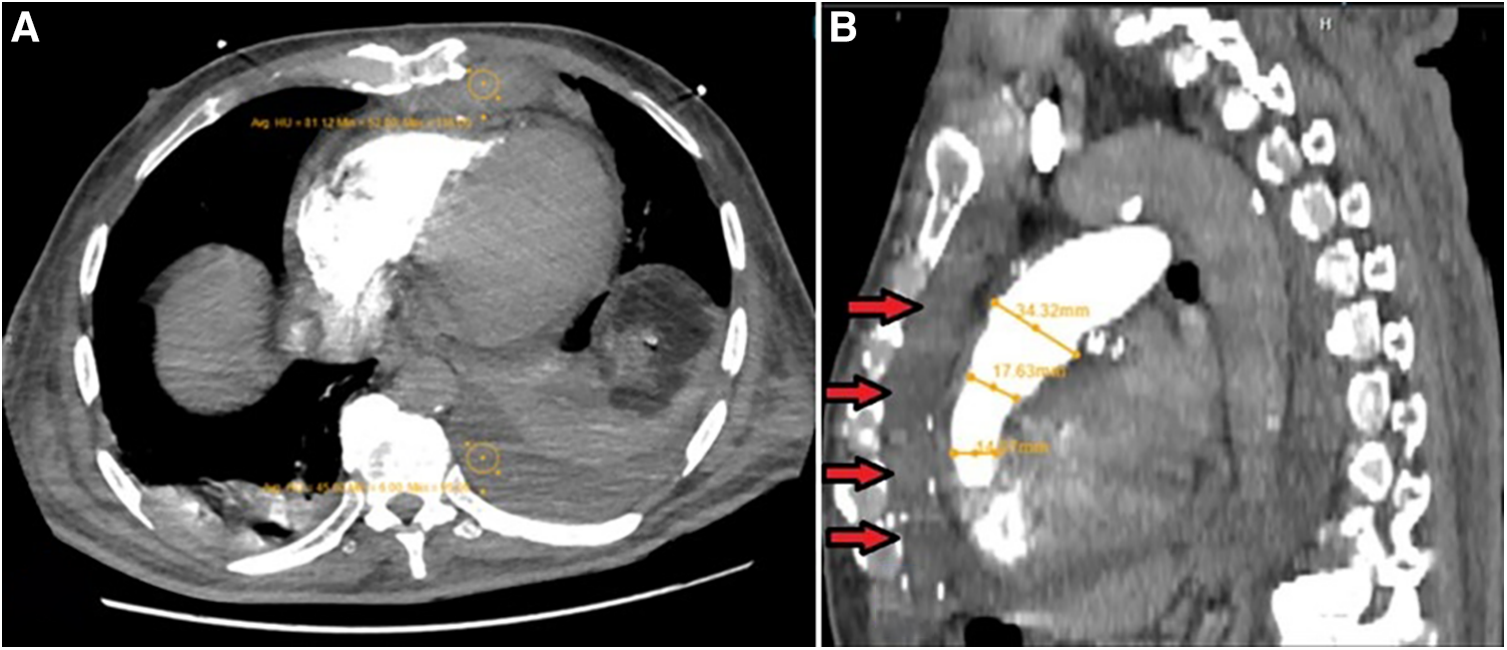
(**A**) Post-contrast axial CT scan image during pulmonary arterial phase, which shows high soft tissue density lesion in the anterior mediastinum with hounsfiled unit (HU) consistent with clotted blood (HU = 81). Also note the presence of mild left-sided bloody pleural effusion (HU = 45), associated with the underlying collapse of the posterior basal segment of the left lower lobe and a lesser degree of segmental atelectasis of the posterior basal segment of the right lower lobe, all secondary to recent CABG operation. (**B**) MPR images show narrowing of RVOT secondary to external pressure effect by retrosternal hematoma (arrows).

After a couple of days, IABP was removed, and the patient was extubated. While serum levels of electrolytes were within the normal range, long QTc remained. Accordingly, IV infusion of magnesium sulfate and lidocaine continued for more following 5 days. Several episodes of sustained and non-sustained TdPs were noted during this period, managed by DC shocks when sustained, resulting in decreased blood pressure. These treatment modalities helped the patient return to sinus rhythm each time. Meanwhile, a bedside transthoracic echocardiography by an expert cardiologist echocardiographer showed a large-size retrosternal hematoma, which extended to the apicolateral of the RV with a compression effect over the RV-free wall. A chest computed tomography (CT) with intravenous contrast was performed for a more accurate evaluation of the hematoma. Post-contrast scan illustrated a high soft tissue density lesion in the anterior mediastinum with Hounsfiled unit (HU) consistent with clotted blood (Figure 3). After retrosternal clot removal by the second surgery, the patient's malignant arrhythmia stopped without any other interventions. Antiarrhythmic drugs stopped and the patient stayed in the hospital for another 2 weeks for close follow-up. Considering in-hospital repeated episodes of TdP before the second surgery, as well as persistent long QTc post-second surgery, he underwent uneventful single chamber implantable cardioverter defibrillator (ICD) implantation. During his 12-month follow-up, ICD analysis showed no high ventricular rate, despite the existence of long QT on several ECGs taken during this follow-up period. Historical care is organized as a timeline (Table 1).

**Table 1:** Timeline of the historical care organized by time.

Date	Day	Clinical signs and symptoms	Imaging or intervention	Results	Physician's decision
24.01.2022	1	Retrosternal pain, nausea, dyspnea	ECG	ST-elevation MI + prolonged QT interval	Advised to undergo emergency coronary angiography
24.01.2022	1	The patient was admitted	Emergent coronary angiography	Stenosis in the terminal end of left main artery + advanced 3VD	CABG was recommended
24.01.2022–03.02.2022	1–11	Admitted to cardiac surgery ward for controlling blood sugar	Serum analysis	Normal serum level of potassium, magnesium, and calcium	Close observation in the ward
24.01.2022–03.02.2022	1–11	Blood sugar was controlled	Echocardiography	Dilated LV + hypokinesia and severe LV dysfunction, EF = 25%, apical aneurysm + apical clot, grade II diastolic dysfunction	CABG with LV aneurysmectomy
03.02.2022	11	Transferred to operation room	Surgery	CABG + aneurysmectomy + clot removal with IABP insertion	Transferred to intensive care unit
05.02.2022–10.02.2022	13–18	Recurrent episodes of TdP, resulting in VF and cardiovascular collapse	ECG	200 J DC shock + 2 gr IV lidocaine + 24 gr magnesium sulfate in 24-h infusion IABP was re-inserted	Follow-up
07.02.2022	15	Hemodynamically stabilized	IABP was removed, extubated	Normal serum level of electrolytes + remained long QT interval	Continue magnesium sulfate and lidocaine infusion for 5 days
05.02.2022–10.02.2022	13–18	Sustained and non-sustained TdP	DC shock + treatment of hypotension	Return to sinus rhythm each time, but relapsed	Echocardiography
10.02.2022	18	Sustained and non-sustained TdP	Echocardiography	Large retrosternal hematoma compressing the free wall of right ventricle	Chest CT
10.02.2022	18	Sustained and non-sustained TdP	Chest CT with contrast	high soft tissue density lesion in the anterior mediastinum with Hounsfiled unit consistent with clotted blood	Second surgery
11.02.2022	19	Admitted to the operation room	Second surgery	Removal of retrosternal clot	Stay in hospital for 2 weeks
23.02.2022	31	Underwent ICD implantation		Uneventful ICD-VR implantation	OPD follow up
25.02.2022	33	Discharged from hospital		Plan ICD analysis and programming	Follow-up for a year
26.02.2023	A year later	OPD visit and ICD analysis every three months over the following year		No high ventricular rate had recorded over the one-year ICD follow up analysis	Follow-up

ECG, electrocardiography; MI, myocardial infarction; 3VD, three-vessel disease; LV, left ventricle; CABG, coronary artery bypass surgery; EF, ejection fraction; IABP, intra-aortic balloon pump; TdP, torsad de point; VF, ventricular fibrillation; CT, computed tomography.

Considering the patient's perspective, he appeared cooperative and established a friendly patient-physician relationship. He had some extent of anxiety, considering the persistant arrhythmia that resulted in longer hospitalization; but after the second surgery that resulted in clinical improvement, the patient's anxiety faded away, and he went home satisfied with the care he received.

## Discussion

Here, we reported a patient with TdP arrhythmia after CABG, resolved by the removal of the retrosternal hematoma. Considering the wide range of factors inducing TdP after CABG, we reported this case to emphasize that the etiology of arrhythmia has to be discovered first, and the treatment should be selected accordingly. Supraventricular arrhythmias, especially atrial fibrillation, are very common after cardiac surgery and affect the prognosis and duration of hospital stay (7, 8). Similarly, ventricular arrhythmias are also observed secondary to MI and/or CABG with several patient- and surgery-related risk factors (9). Finding the cause of arrhythmia formation can help the physician to treat it before it leads to fatal complications.

The type of arrhythmia in the patient reported here was TdP, a rare malignant arrhythmia that is a specific form of polymorphic VT occurring in the context of QT prolongation (10). Interestingly, our patient presented with long QTc at the time of referral (before the surgery). A review of all cases (available in the literature) suggested cardiac surgery and craniotomies accounting for 40% of all cases with perioprative TdP (11). Reports suggest that TdP is probably not the direct effect of CABG, but related to CABG complications (12–14), such as ischemia (due to graft failure) (12) or co-administartion of Q-T prolonging drugs (like amiodarone) in the perioperative period (13, 15). It is important to remember that prolonged QTc is multifactorial and can occur by genetic and inhertited causes, as well (14). But, the exact cause of pre-surgical QT prolongation was not clarified in our patient, because the genetic testing was not done (due to the patient's financial inability); also, the patient did not take any Q-T prolonging drugs. Q-T prolonging drugs are found responsible for at least one-third of cases with perioperative TdP (11); some patients who develop drug-induced TdP are silent carriers of gene mutations related to prolonged QTc (16). While TdP development as a result of medications (like terfenadine and cisapride) is a cause of withdrawal, many other medications (like amiodarone and ranolazine) are still on the market, as they mainly cause prolonged QTc, but rarely TdP. Bearing in mind that the cumulative effect of two QTc-prolonging agents increases the risk in the patient, it is necessary to consider drug interactions (mediated through cytochrome P540) in the perioperative period (17, 18). Other risk factors of prolonged QTc include female sex, higher age, electrolyte abnormalities, anorexia nervosa, heart conditions (such as bradycardia, left ventricular dysfunction, heart failure, mitral valve prolapse, and MI), and other medical conditions (like renal/hepatic dysfunction, hypokalemia, hypoglycemia, hypertension, diabetes mellitus, hypothyroidism, pituitary insufficiency, injury to the central nervous system, malnutrition, and obesity) (18–20). Uncommon causes, like coronary atherosclerotic plaque rupture after thoracic trauma, have also been reported (21). There are also reports of patients with subdural hematoma (following head trauma), found to have TdP during admission (22–24). Some have also reported TdP as the presenting sign of cerebral hemorrhage (25). Therefore, it is necessary to pay attention to the multiple risk factors of TdP development and prevent co-adminstration of factors that increase the risk of TdP development and consider cases who are more susceptible to this arrhythmia, in order to diagnose it at an early stage and implement therapeutic strategies before fatal complications occur.

The case presented here had none of the above-mentioned causes; but, another rare postoperative complication was observed that we speculate it as the main cause of TdP formation. In this case, imaging investigations (accurate examinations by echocardiography and CT scan) showed a post-CABG retrosternal hematoma, causing localized tamponade. Hematoma, caused by the leaking of blood from an aneurysmectomy/aneurysmorrhaphy site, is listed among common postoperative complications of CABG; but not as a cause of post-CABG TdP. Hematoma, alone, is an important complication, as it can rarely compress the heart and arteries and may even cause superior vena cava syndrome (26) and tamponade, which lead to shock and death in the patients (27). Therefore, diagnosis is essential; previous studies have suggested CT scan as an accurate diagnostic tool for post-CABG epicardial and retrosternal hematoma formation (28, 29). We could also detect the details of the developed hematoma by CT scan successfully. This case shows the necessity to closely observe any patient who develops hypotension after CABG. Fortunately, our case was diagnosed appropriately and saved by appropriate treatment.

The need for re-operation has been reported previously in patients with post-CABG hematoma (29–34). We also removed the hematoma with a second surgery. A notable issue in our patient was the resolution of TdP after this (second) surgery, which suggests that the development of TdP after the first surgery could have been caused by the pressure effect of hematoma on the heart or small coronary arteries, which could lead to ischemia. We did not observe a similar case reporting an association between retrosternal hematoma and medically intractable TdP. This finding [resolution of arrhythmia after the second surgery (hematoma removal)] in our case suggests a “cause and effect” relationship between hematoma and drug-resistant TdP, which is of great significance, considering the challenging treatment of TdP (35). Therefore, post-CABG hematoma should also be considered in cases who have sustained or recurrent TdP arrhythmia.

Other rare points documented in the present case report include the time and site of the hematoma. Development of hematoma at the early postoperative phase has been only reported in a few cases before (32, 33); most of the cases with true hematoma are reported at the delayed phase after CABG (29–31), while most of the early cases are found to be pseudoaneurysms. Therefore, hematoma should be considered in the differential diagnoses of patients with early postoperative complications, as well. Also, the common sites of post-CABG hematoma are pericardial (29, 34) and very rarely retrosternal (like our case). The concurrency of retrosternal hematoma and TdP in our patient was a rare finding, and the resolution of this malignant arrhythmia by removal of hematoma was an important finding that has to be noted in future studies.

## Conclusion

Retrosternal hematoma can form after CABG as an early postoperative complication. Arrhythmias may also occur or aggregate as a result of surgery. Co-ocurence of retrosternal hematoma and arrhythmia (drug-resistant TdP) after CABG has been noted in the present case, as a rare finding. Bearing in mind that TdP is a malignant arrhythmia and may result in fatal complications, it is important to find the exact cause of TdP occurrence, among the multiple risk factors, in order to find an appropriate treatment; many cases are refractory to conventional treatments. In the case presented here, the resolution of both post-CABG complications after the second surgery, removal of hematoma, suggested the possibility of a “cause and effect” relationship between hematoma and TdP. This finding has not been reported previously, and our finding suggest that physicians should consider surgical removal of hematoma for the treatment of similar cases, who develop resistant TdP. However, further studies are required to confirm this finding.

## Data Availability

The raw data supporting the conclusions of this article will be made available by the authors, without undue reservation.

## Supplementary material

Supplementary Figure S1 The post-CABG ECG of the patient in the intensive care unit, which illustrates normal sinus rhythm with low voltage QRS, and bigeminal ventricular extrasystole (PVCs) with short coupling interval (R on T wave).

## References

[1] Jayaraj JC Davatyan K Subramanian S Priya J. Epidemiology of myocardial infarction. In: Pamukçu B, editors. Myocardial Infarction. 3rd ed. Rijeka: IntechOpen (2018). 10.5772/intechopen.74768

[2] Lawton JS Tamis-Holland JE Bangalore S Bates ER Beckie TM Bischoff JM 2021 ACC/AHA/SCAI guideline for coronary artery revascularization: executive summary: a report of the American College of Cardiology/American Heart Association Joint Committee on clinical practice guidelines. J Am Coll Cardiol. (2022) 79:197–215. 10.1016/j.jacc.2021.09.005 34895951

[3] Durko AP Budde RP Geleijnse ML Kappetein AP. Recognition, assessment and management of the mechanical complications of acute myocardial infarction. Heart. (2018) 104:1216–23. 10.1136/heartjnl-2017-311473 29146624

[4] Jawitz OK Gulack BC Brennan JM Thibault DP Wang A O'Brien SM Association of postoperative complications and outcomes following coronary artery bypass grafting. Am Heart J. (2020) 222:220–8. 10.1016/j.ahj.2020.02.002 32105988 PMC7085463

[5] Chiriac L Rosulescu R. Arrhythmias and conduction disturbances after coronary artery bypass graft surgery. In: Ţintoiu I Underwood M Cook S Kitabata H Abbas A, editors. Coronary Graft Failure. Switzerland: Springer, Cham (2016). p. 167–74.

[6] Halkin A Roth A Lurie I Fish R Belhassen B Viskin S. Pause-dependent torsade de pointes following acute myocardial infarction: a variant of the acquired long QT syndrome. J Am Coll Cardiol. (2001) 38:1168–74. 10.1016/s0735-1097(01)01468-1 11583899

[7] Lee G Sanders P Kalman JM. Catheter ablation of atrial arrhythmias: state of the art. Lancet. (2012) 380:1509–19. 10.1016/s0140-6736(12)61463-9 23101718

[8] Egbe AC Connolly HM McLeod CJ Ammash NM Niaz T Yogeswaran V Thrombotic and embolic complications associated with atrial arrhythmia after fontan operation: role of prophylactic therapy. J Am Coll Cardiol. (2016) 68:1312–9. 10.1016/j.jacc.2016.06.056 27634123

[9] Peretto G Durante A Limite LR Cianflone D. Postoperative arrhythmias after cardiac surgery: incidence, risk factors, and therapeutic management. Cardiol Res Pract. (2014) 2014:615987. 10.1155/2014/615987 24511410 PMC3912619

[10] Neira V Enriquez A Simpson C Baranchuk A. Update on long QT syndrome. J Cardiovasc Electrophysiol. (2019) 30:3068–78. 10.1111/jce.14227 31596038

[11] Johnston J Pal S Nagele P. Perioperative torsade de pointes: a systematic review of published case reports. Anesth Analg. (2013) 117:559–64. 10.1213/ANE.0b013e318290c380 23744954 PMC3750104

[12] Iguina MM Smithson S Danckers M. Incessant refractory polymorphic ventricular tachycardia after coronary artery bypass graft. Cureus. (2021) 13:e12752. 10.7759/cureus.12752 33643727 PMC7886165

[13] Pasley T Gillham M. The effect of cardiac surgery on the QT interval. Are patients at higher risk of torsades de pointes post cardiac surgery? Heart Lung Circ. (2014) 23:e16–e7. 10.1016/j.hlc.2014.04.16823948290

[14] William J Shembrey J Quine E Perrin M Ridley D Parameswaran R Polymorphic ventricular tachycardia storm after coronary artery bypass graft surgery: a form of ‘angry purkinje syndrome’. Heart Lung Circ. (2023) 32:986–92. 10.1016/j.hlc.2023.04.298 37210317

[15] Kodaka M Mori T Ichikawa J Ando K Komori M. Refractory ventricular arrhythmias during aortic valve replacement and cardiac artery bypass requiring 16 attempts of electrical cardioversion: a case report. JA Clin Rep. (2020) 6:60. 10.1186/s40981-020-00369-w 32783130 PMC7419391

[16] Wallace E Howard L Liu M O’Brien T Ward D Shen S Long QT syndrome: genetics and future perspective. Pediatr Cardiol. (2019) 40:1419–30. 10.1007/s00246-019-02151-x 31440766 PMC6785594

[17] Schwartz PJ Woosley RL. Predicting the unpredictable: drug-induced QT prolongation and torsades de pointes. J Am Coll Cardiol. (2016) 67:1639–50. 10.1016/j.jacc.2015.12.063 27150690

[18] Beach SR Celano CM Noseworthy PA Januzzi JL Huffman JC. QTc prolongation, torsades de pointes, and psychotropic medications. Psychosomatics. (2013) 54:1–13. 10.1016/j.psym.2012.11.001 23295003

[19] Niimi N Yuki K Zaleski K. Long QT syndrome and perioperative torsades de pointes: what the anesthesiologist should know. J Cardiothorac Vasc Anesth. (2022) 36:286–302. 10.1053/j.jvca.2020.12.011 33495078

[20] Iragavarapu T Krishna K. Torsades De pointes complicating acute myocardial infarction, twisting the prognosis. Indian Journal of Clinical Cardiology. (2023) 4:208–14. 10.1177/26324636231190247

[21] Gowdak LHW Bittencourt MS Rochitte CE Dallan LAO César LAM. Coronary atherosclerotic plaque rupture following thoracic trauma: an uncommon cause of angina and ventricular tachycardia (“torsade de pointes”). Clinics (Sao Paulo). (2011) 66:1291–3. 10.1590/s1807-59322011000700029 21876990 PMC3148480

[22] McCoy C Miller JM Tanawuttiwat T. A woman with recurrent torsade de pointes. JAMA Cardiol. (2023) 8:400–1. 10.1001/jamacardio.2022.5094 36652237

[23] Bottigliero D Monaco I Santacroce R Casavecchia G Correale M Guastafierro F Novel AKAP9 mutation and long QT syndrome in a patient with torsades des pointes. J Interv Card Electrophysiol. (2019) 56:171–2. 10.1007/s10840-019-00606-y 31418098

[24] Carpenter K Ahmed I Boland T. Torsades de pointes as a late complication of subarachnoid hemorrhage (P2. 287). Neurology. (2017) 88:P2.287. 10.1212/WNL.88.16_supplement.P2.287

[25] Wang K-C Chen S-Y. Cerebellar hemorrhage presented as torsades de pointes. Tungs' Med J. (2022) 16:34–6. 10.53106/207135922022061601007

[26] Ibrahim R Yadav S Waqar S Hermann JR Sarwar A Shah S. Superior vena Cava syndrome due to right anterior mediastinal hematoma: a case report. Cureus. (2022) 14:e26994. 10.7759/cureus.26994 35989818 PMC9385572

[27] Kanzaki H. Invisible hematoma causing shock after open-heart surgery: localized cardiac tamponade. J Cardiol Cases. (2014) 9:243–4. 10.1016/j.jccase.2014.02.002 30534337 PMC6278562

[28] Helmy IM Asbeutah AM ElFiki IM Arafa OE. A pictorial review on the role of 64-slice HD MDCT in detecting post CABG cardiothoracic complications. Open Journal of Thoracic Surgery. (2014) 4:48–58. 10.4236/ojts.2014.42011

[29] Floerchinger B Camboni D Schopka S Kolat P Hilker M Schmid C. Delayed cardiac tamponade after open heart surgery-is supplemental CT imaging reasonable? J Cardiothorac Surg. (2013) 8:158. 10.1186/1749-8090-8-158 23800191 PMC3698060

[30] Ohira S Matsushita T Masuda S. Late mediastinal hematoma presenting cardiac tamponade after re-do off-pump coronary artery bypass grafting in octogenarian; report of a case. Kyobu Geka. (2012) 65:1003–5. 23023547

[31] Yamamoto T Takahashi K Tsukioka K Kono T. Successful repair of chronic expanding hematoma after coronary artery bypass grafting by lower partial sternotomy approach; report of a case. Kyobu Geka. (2017) 70:863–6. 28894061

[32] Ananthasubramaniam K Jaffery Z. Postoperative right atrial compression by extracardiac hematoma: transesophageal echocardiographic diagnosis in the critically ill patient. Echocardiography. (2007) 24:661–3. 10.1111/j.1540-8175.2007.00445.x 17584209

[33] Grumann A Baretto L Dugard A Morera P Cornu E Amiel J-B Localized cardiac tamponade after open-heart surgery. Ann Thorac Cardiovasc Surg. (2012) 18:524–9. 10.5761/atcs.oa.11.01855 22785553

[34] Khan Z Srour K Khan M Moustafa A Javaid T. Anterior mediastinal hematoma and right sided hemothorax from leaking saphenous vein right coronary artery bypass graft aneurysm due to incomplete coiling masquerading as right lower lobe pneumonia. Am J Respir Crit Care Med. (2018) 197:A3445. 10.1007/s11739-018-1847-5 29619768

[35] Thomas SH Behr ER. Pharmacological treatment of acquired QT prolongation and torsades de pointes. Br J Clin Pharmacol. (2016) 81:420–7. 10.1111/bcp.12726 26183037 PMC4767204

