# Supplementary figures and images for: Retrosternal hematoma causing torsade de pointes after coronary artery bypass graft surgery; a case report
Source: Front Cardiovasc Med. 2024 May 20;11:1331873. doi: 10.3389/fcvm.2024.1331873 (PMC11144857; doi:10.3389/fcvm.2024.1331873)

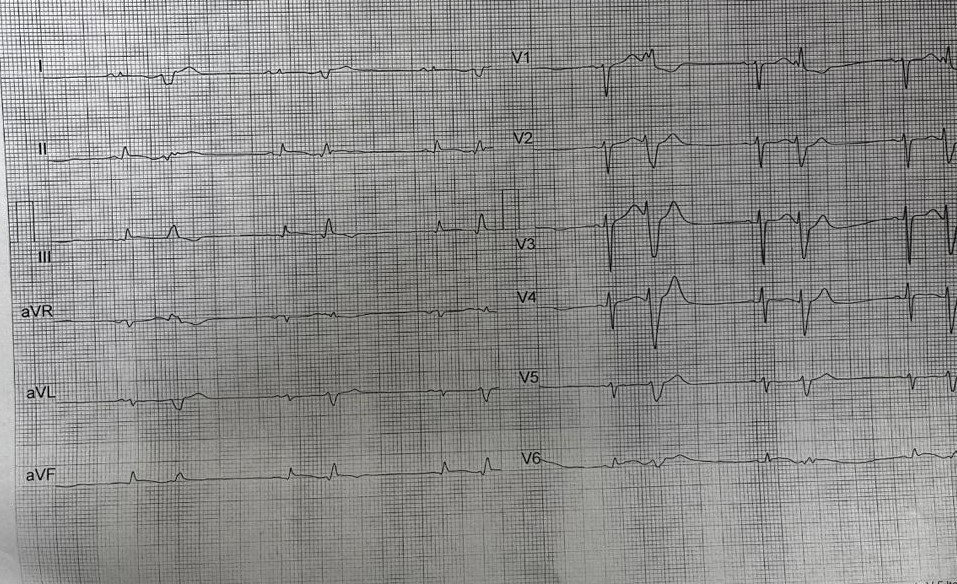

Supplement: Supplementary Figure S1 — The post-CABG ECG of the patient in the intensive care unit, which illustrates normal sinus rhythm with low voltage QRS, and bigeminal ventricular extrasystole (PVCs) with short coupling interval (R on T wave). [file Image1.jpeg]
